# A novel potential primary method for quantification of enantiomers by high performance liquid chromatography-circular dichroism

**DOI:** 10.1038/s41598-018-25682-4

**Published:** 2018-05-09

**Authors:** Yi Luo, Liqing Wu, Bin Yang, Youxun Jin, Kangle Zheng, Zhangjing He

**Affiliations:** 1 National Institute of Metrology, P.R. China, Beijing, China; 2 Beijing University of Chemical Technology, Beijing, China

## Abstract

Primary methods play an important role in metrology. They can be used for the value assignment of certified reference materials, enabling the accuracy and comparability of the measurement. A novel potential primary method for enantiomer quantitation based on high-performance liquid chromatography-circular dichroism is described using L-phenylalanine as an example. The optimal quantitation range of L-Phe was from 0.1 mg/g to 1.2 mg/g, where both the relative bias and method variance were lower than 1%. The LOD and LOQ were 4 μg/g and 30 μg/g, respectively. The proposed method was also applied to the determination of the mass fraction of pure porcine insulin in solid. The average mass fraction obtained was 0.922 g/g with a RSD of 1.5%, and the associated relative uncertainty is 3.8% (*k* = 2), which agreed well with that obtained from the traditional isotope dilution mass spectrometry method. The LOD and LOQ for insulin quantitation were found to be 0.12 mg/g and 0.44 mg/g, respectively. The proposed method can be entirely described and understood by equations and a complete uncertainty statement can be defined in SI units.Therefore, it may be a potential primary method useful for the quantification of chiral compounds and proteins, and a supplementary method to the traditional isotope dilution mass spectrometry approach.

## Introduction

Proteins are a group of large bio-molecules and consist of one or more chains of amino acid residues. Proteins play an important role in the regulation of all types of organism functions. Most bio-markers are proteins and are used as bio-pharmaceutical targets, diagnostic markers, and food safety inspection markers. Therefore, an accurate and comparable result of protein measurement is necessary for effective and correct disease treatment and safety management. To ensure the measurement is accurate and comparable, generally a traceability chain should be established and certified reference materials (CRMs)^1^ should be developed for value transferring. National Metrology Institutes are responsible for primary method development so as to maintain the traceability chain and accurately assign the values to the CRMs.

The term “primary method” defined by Consultative Committee for Amount of Substance (CCQM) in 1998 represents a method having the highest metrological qualities, whose operation can be completely described and understood^2^, for which a complete uncertainty statement can be written down in terms of SI units. CCQM notes that a primary method plays a fundamental role in the application of metrology to chemistry. The primary methods can be divided into two categories: a primary direct method and a primary ratio method^2^. The primary direct method measures the value of an unknown without reference to a standard of the same quantity while the primary ratio method measures the value of a ratio of an unknown to a standard of the same quantity and its operation must be completely described by a measurement equation. Several measurement methods have been identified as potential primary methods by CCQM, including isotope dilution mass spectrometry (IDMS), coulometry, gravimetry (with gas mixtures or gravimetric analysis), titrimetry and colligative methods^2^. However, most of these primary methods are not suitable for protein analysis except IDMS.

As one of the potential primary methods, IDMS are widely used for quantification of pure proteins as well as proteins in a matrix. Generally, a stable isotope labelled amino acid, peptide or protein is used as the internal standard, and mixed with the sample. Then the mixture is hydrolysed to amino acids or digested to peptides. The amino acid or the signature peptide is separated by HPLC and then analysed by mass spectrometry. The ratio of the compound to that of the labelled one is measured and the mass fraction of the pure protein or the protein in the matrix is calculated based on the ratios and the weighing. Amino acid-based IDMS allows detection of a protein using hydrolysis in the presence of an isotope labelled amino acid as the internal standard. After complete hydrolysis, the amount of protein can be calculated from the amount of amino acids. The expanded uncertainty of amino acid-based IDMS was reported to be from 1.8% to 6%^3–6^. Peptide-based IDMS has been successfully used in the quantification of insulin chain B^7^, lysozyme^8^, human serum albumin^9^, human growth hormone^10^, and prostate-specific antigen^11^ among others.

In recent years, some new potential primary methods have been proposed for protein quantitation, such as mass balance, q-NMR, and isotope dilution surface enhanced Raman spectrometry (HPLC-ID-SERS). However, there are still many disadvantages to the new proposed methods. The mass balance method is only suitable for a large amount of solid material with high purity^12,13^. Identification and quantification of each impurity is difficult to achieve. The q-NMR method has been successfully applied for the quantification of a pure peptide, but it is less suitable for proteins due to peak overlap^14,15^. The ID-SERS method is a type of potential primary ratio method and is similar to the IDMS method. However, its application is limited because of the lack of on-line separation technology combined with Raman spectrometry. Therefore, a new universal primary method for protein quantitation could be a useful supplement to the current IDMS method. Cross-validation of the different potential primary methods would benefit the confidence of the measurement result.

**Figure 1:**
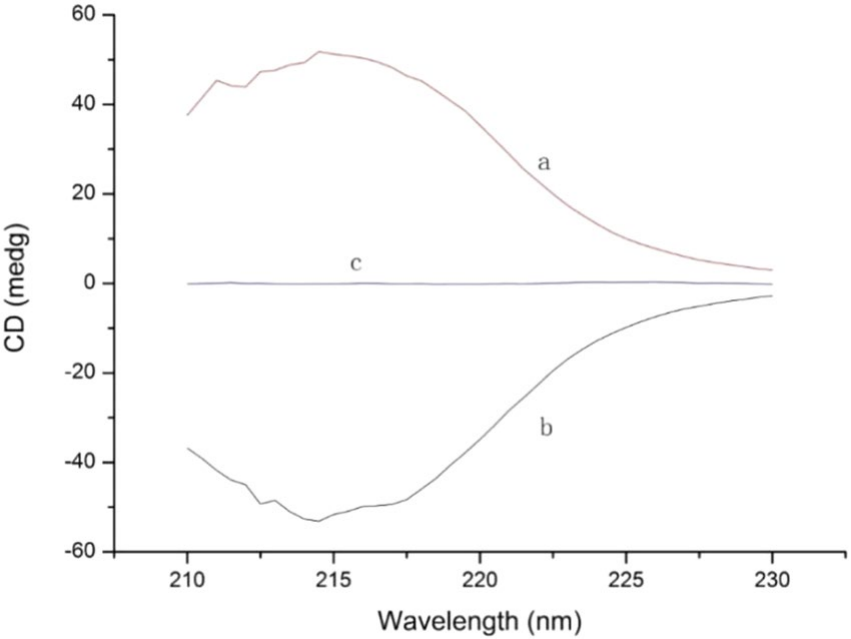
The circular dichroism spectra of L- and D-phenylalanine. (a and b) Are the circular dichroism spectra of the same amount of L- and D-phenylalanine, respectively. They have an approximately equal but opposite signal. (c) Is the spectrum from a mixture (racemate) of the same amount of L- and D-phenylalanine).

Circular dichroism (CD), a type of dichroism involving circularly polarized light, is the differential absorption of left- and right-handed light. The absorbance of left- and right-handed light are different for a chiral compound, which results in a signal in the CD spectrum^16^. Traditionally, CD spectrums are used for optical purity assessment, enantiomeric excess measurement, and estimation of protein secondary structures^17–19^. Generally, a pair of enantiomers has mirrored CD spectrums as shown in Fig. 1. It shows the equal but opposite CD signal of the individual enantiomers indicating that if equal amounts of enantiomers are mixed (racemate), the CD signal will be zero or at the baseline. Therefore, the CD spectrometer acts as a balance and the enantiomer is like the poise weight. One enantiomer can be used to measure the related paired enantiomer by CD spectrometry.

A novel potential primary method based on high-performance liquid chromatography-circular dichroism (HPLC-CD) technology is described herein. The measurement principles are discussed thoroughly and the methods are optimized for L-phenylalanine quantitation. Its application for porcine insulin quantitation is also investigated and compared with that from the IDMS method. The proposed method can be a useful supplement to the IDMS method and benefit the measurement confidence of the quantitation results of enantiomers and proteins.

## Methods

### Instruments and reagents

The electronic balances, Mettler Toledo XP56 (0.001 mg) and XS205 (0.01 mg), were purchased from Mettler Toledo (Zurich, Switzerland). The high-performance liquid chromatography system (HPLC, Aglient 1260 Infinity), equipped with an auto sampler, quaternary pump, column compartment, and DAD detector (Agilent 1290 Infinity), were purchased from Agilent Technologies (Santa Clara, CA, USA). The AD converter 1200 Infinity Universal Interface Box II was purchased from Agilent Technologies. The Waters Accq-Tag column (2.1 mm ×150 mm, 1.7 μm) and Waters UPLC HSS T3 (2.1 mm ×100 mm, 1.8 μm) columns were purchased from Waters (Milford, MA, USA) and the Daicel CROWNPAK CR+ column (4.0 mm ×150 mm, 5 μm) was purchased from Daicel Corporation (Tokyo, Japan). The circular dichroism spectrometer (JASCO J-815) was purchased from Jasco Corporation (Tokyo, Japan). The ultrasonic cleaning device (KQ-500DE) was purchased from KunShan Ultrasonic Instrument Corporation (Kunshan, China). The oven was purchased from Memmert GmbH + Corporation (Schwabach, Germany). The Milli-Q ultrapure water system was purchased from Merck KGaA (Darmstadt, Germany).

D-phenylalanine (D-Phe) was purchased from Sinopharm Chemical Reagent Co., Ltd (Shanghai, China). The L-phenylalanine (L-Phe) CRM, L-glycine CRM and porcine insulin (pINS) CRMs were from the National Institute of Metrology (P.R. China). L-leucine, L-valine, L-proline, L-isoleucine, L-arginine and L-methionine were purchased from Fluka, Merck KGaA (Darmstadt, Germany). L-glutamic acid, L-serine, L-histidine, L-threonine, L-aspartic acid, L-lysine and L-alanine were purchased from Solarbio (Beijing, China). Acetonitrile was purchased from Fisher Scientific (Waltham, MA, USA). Hydrochloric acid (A.R. grade) and perchloric acid (A.R. grade) were purchased from Beijing Chemworks (Beijing, China). The deionized water was prepared with the Milli-Q system.

### Preparation of samples and standards

The solid L-Phe CRM and D-Phe were dissolved in deionized water to a final concentration of 1 mg/g. Then the stock solution was diluted to 0.005, 0.01, 0.02, 0.03, 0.04, 0.05, 0.06, 0.08, 0.1, 0.3, 0.5, 0.8, and 1 mg/g to prepare the standards. A similar series of dilutions was made for the D-Phe solutions.

For some analyses, a series of concentrates of L-Phe were mixed with 0.05 mg/g of D-Phe in the same amount as the fixed concentrate mixture. Different amounts of L-Phe with D-Phe in almost the same concentration were prepared as the variable concentrate mixture. Hydrochloric acid was diluted from 12 mol/L to 8 mol/L in deionized water for hydrolysis and 8 mol/L hydrochloric acid was diluted to 0.1 mol/L in deionized water as the solvent for the pINS.

The amino acid mixture, containing L-leucine, L-valine, L-proline, L-isoleucine, L-phenylalanine, L-alanine, L-lysine, L-threonine, L-aspartic acid, L-histidine, L-methionine, L-arginine, L-glutamic acid, L-glycine and L-serine, was prepared by solid pure amino acids (AAs) and deionized water, with a final concentration of 4 mg/g.

### Optimization of the CD parameters

A L-Phe solution (0.1 mg/g) was added to a cuvette and used to optimize the parameters for CD detection. The CD signal intensities were compared using different wavelengths, band widths, and digital integration times (DIT) and a 0.1 mg/g L-Phe solution. The wavelength range was optimized using the Spectra software to be 200 nm to 300 nm. The DIT was optimized using the optimal detection wavelength. The CD spectrum was scanned with a DIT from 1 to 32 s using the Spectra software that comes with the CD instrument. The optimized parameters provided the maximum CD intensity.

### Optical purity examination of the D-Phe

Because D-Phe was used as the internal standard, the existence of L-Phe in the D-Phe will lead to a bias of the quantitation result. Therefore, the D-Phe was examined to ensure there was no L-Phe in it. Several enantio separation^20,21^ methods for D/L-Phe using HPLC have been described before. In this study, a chiral stationary phase (CSP) was chosen to estimate the purity of the D-Phe. To separate the L-Phe from the D-Phe, a chiral column (CROWNPAK CR+, 4.0 mm×150 mm) was used. A perchloric acid aqueous solution at a pH of 1.55 was used as the mobile phase with a flow rate of 0.8 mL/min. The injection volume was 10 μL and the detection wavelength was 200 nm.

### Optimization of the HPLC separation conditions

HPLC-CD was used to quantify the protein in solution and therefore, the protein was first hydrolysed into an amino acid mixture. Then the L-Phe was quantified by HPLC-CD and the value was used to calculate the concentration of protein according to the amino acid composition. Therefore, it is important to ensure the L-Phe was separated from other amino acids. To optimize the HPLC separation conditions, we fixed the injection volume, detection wavelength and flow rate, and the column as well as the mobile phase were optimized. The injection volume was 10 μL and the detection wavelength was 216.5 nm. The flow rate was 0.2 mL/min. A Waters UPLC HSS T3 column was used for the separation with a mobile phase A of deionized water and phase B of acetonitrile. The different ratios of A:B (100:0, 98:2, 95:5, 90:10, 80:20) were compared for the separation of Phe from the AA mixture.

### Application of the method to pINS quantitation

Each pINS sample was prepared on the electronic balance with 0.1 mol/L hydrochloric acid to a final concentration of 12 mg/g. A 200 μL aliquot of the sample solution was weighed and added into the ampoule with 50 μL of D-Phe solution at 0.2 mg/g as the internal standard. Then 750 μL of 8 mol/L hydrochloric acid was weighed and added into the same ampoule. They were mixed thoroughly and then the oxygen in the ampoule was removed by flushing with nitrogen before it was sealed. The hydrolysis was carried out at (110.0 ± 0.5) °C for 48 h. The sample was analysed directly in the HPLC-CD after hydrolysis. The different ratios of A:B (100:0, 98:2, 95:5) were compared for the separation of L-Phe from the hydrolysate mixture of pINS.

To evaluate the precision of the method, three different samples were hydrolysed and analysed six times each to calculate the concentration of pINS. Also, the intra-day and inter-day results of the method were evaluated.

## Results and Discussion

### Measurement principle

The CD signal is based on the measurement of the differential absorption of the left- and right-handed light of a chiral compound, which can be expressed by the following equation:

1 $$I={\rm{\Delta }}A={A}_{L}-{A}_{D}=({\varepsilon }_{L}-{\varepsilon }_{D})cl$$

where:

*I* and Δ*A* are the CD signal and the difference between the absorbance of left circularly polarized and right circularly polarized light, respectively;

${\varepsilon }_{L}$ and ${\varepsilon }_{D}$ are the molar extinction coefficients of left circularly polarized and right circularly polarized light, respectively;

*c* is the molar concentration;

*l* is the path length.

For a pair of simple chiral compounds, such as L-Phe and D-Phe, the following equations exist:

2 $${\varepsilon }_{L}=-\,{\varepsilon ^{\prime} }_{L}$$

3 $${\varepsilon }_{D}=-\,{\varepsilon ^{\prime} }_{D}$$

where:

${\varepsilon }_{L}$ and ${\varepsilon }_{D}$ are the molar extinction coefficients of left circularly polarized and right circularly polarized light of the L-type chiral compound, respectively;

${\varepsilon ^{\prime} }_{L}$ and ${\varepsilon ^{\prime} }_{D}$ are the molar extinction coefficients of left circularly polarized and right circularly polarized light of the D-type chiral compound, respectively.

Based on formula (1) above, the following equations can be obtained:

4 $${I}_{L}=({\varepsilon }_{L}-{\varepsilon }_{D}){c}_{L}l$$

5 $${I}_{D}=({\varepsilon ^{\prime} }_{L}-{\varepsilon ^{\prime} }_{D}){c}_{D}l=-\,({\varepsilon }_{L}-{\varepsilon }_{D}){c}_{D}l$$

If the L-type compound is mixed with the D-type compound, the CD signal intensity can be calculated from formula (6), which can be also derived by combining equations 4 and 5:

6 $$I={I}_{L}+{I}_{D}=K({c}_{L}-{c}_{D})$$

where:

*I* is the CD signal intensity of the mixture;

*K* is $({\varepsilon }_{L}-{\varepsilon }_{D})l$ and is a constant.

When the concentration of the L-type compound is equal to that of the D-type compound, they will give the equal but opposite CD signal. Thus, when the same amount of L-type is mixed with D-type compound, the CD signal will be zero. Therefore, the CD spectrometer is just like a balance and the chiral compounds act as the poise weights. If the “weight” of the “poise weight” is the same, a zero will be shown on the “balance”.

The CD based measurement can be applied to different experimental approaches.

#### Approach 1

In this approach, one chiral compound is used to directly measure its enantiomer. For example, L-Phe in solution is quantified using a known amount of D-Phe CRM. Therefore, this approach is a potential type of primary direct method. A series of known amounts of D-type compounds are added into samples that contain the L-type compound. The CD signal is acquired for the individual mixtures. A zero CD signal, based on formula (6), indicates the concentration of *c*_L_ is equal to *c*_D_. This approach is a type of direct measurement approach.

#### Approach 2

In this approach, one chiral compound is used as the internal standard to measure its enantiomer, and the same reference to a standard of the quantity being measured is required. Therefore, it is a type of ratio measurement.

An L-type compound measurement is used as an example for illustration purposes. When single point calibration is used, the L-type compound CRM is used to prepare the calibrator solution, and then the pure D-type compound is used as the internal standard and added into the calibrator solution. The concentrations of the L- and D-type compounds in the standard mixture are assumed to be *c*_LSTD_ and *c*_D1_, respectively, and the CD signal of the standard mixture is assumed to be *I*_std_. Similarly, the D-type compound is also used as the internal standard and added into the sample containing the L-type compound. The concentrations of the L- and D-type compounds in the sample mixture are assumed to be *c*_L_ and *c*_D2_, respectively, and the CD signal of the standard mixture is assumed to be *I*. Therefore, formulas (7 and 8) can be derived based on formula (6):

7 $${I}_{{\rm{STD}}}=K({c}_{LSTD}-{c}_{D1})$$

8 $$I=K({c}_{L}-{c}_{D2})$$

Solving equations 7 and 8, *c*_L_ can be calculated by formula (9):

9 $${c}_{L}=\frac{I}{{I}_{STD}}({c}_{LSTD}-{c}_{D1})+{c}_{D2}$$

However, when the CD spectrometer is not calibrated properly, an offset exists and the CD signal will not be exactly zero when the amounts of the chiral compounds are equal. In this situation, bracket calibration can be used. When the bracket calibration method is applied, a lower standard and higher standard are needed. The concentration of the L- and D-type compounds in the lower and higher standards are assumed to be *c*_L1_ and *c*_D1_, *c*_L2_ and *c*_D2_, respectively, and the CD signals are assumed to be *I*_1_ and *I*_2_, respectively. The concentrations of the L- and D-type compounds in the sample mixture are assumed to be *c*_L_ and *c*_D_, respectively, and the CD signal of the standard mixture is assumed to be *I*. Therefore, formulas (10–12) can be derived based on formula (6), where b is the offset of the CD signal.

10 $${I}_{{\rm{1}}}=K({c}_{L1}-{c}_{D1})+b$$

11 $${I}_{2}=K({c}_{L2}-{c}_{D2})+b$$

12 $$I=K({c}_{L}-{c}_{D})+b$$

The *K* and *b* can be solved from equations 10 and 11, and then they are used in equation 12 to calculate *c*_L_, which is shown in equation 13.

13 $${c}_{L}=\frac{({I}_{2}-I)({c}_{L1}-{c}_{D1})+(I-{I}_{1})({c}_{L2}-{c}_{D2})}{({I}_{2}-{I}_{1})}+{c}_{D}$$

### Establishment of the HPLC-CD equipment

Only a few commercial CD detectors are available in the market and most are from JASCO^22–25^. However, the typical detection wavelength range and wavelength accuracy of the commercial CD detectors are 220 to 420 nm and 5 nm, respectively. Compared with the J-815 CD spectrometer with values of 166 to 1100 nm and 0.1 nm, the performance of the commercial CD detectors is poor. Also, the maximum CD signal intensity of most amino acids is achieved using a wavelength range of 210 nm to 220 nm. Therefore, a commercial CD detector was not suitable for this work and it was necessary to design a LC-CD system with a wavelength range of 210 nm to 220 nm. A J-815 CD spectrometer from JASCO was combined with an Agilent 1260 LC. For the flow path system, a commercial flow cell from JASCO was installed on the J-815 CD spectrometer. The inlet of the flow cell was connected with the outlet of the HPLC DAD detector by PEEK tubing. To synchronize the trigger signal of injection from the LC, a cable was used to connect the trigger input and output ports. The CD intensity analogue signal was also introduced from the TP3 and TP6 ports of the CD spectrometer to an AD converter from Agilent, which allows the Agilent workstation to collect and record the CD signal. The CD signal can be also collected and recorded by the Time Course software provided by JASCO. The absorbance of CD spectrometer was calibrated with neutral density filter CRMs (GBW(E)130225). The CD signal was firstly calibrated with commercial calibrator solution (Sodium Camphor Sulfonate) provided by JASCO company. Then the zero signal was validated by commercial racemic phenylalanine solution as well as prepared solution with equal amount of L- and D-phenylalanine (Fig. 1).

### Optimization of the CD parameters

**Figure 2:**
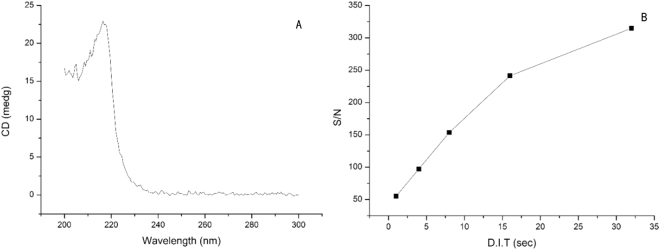
(**A**) Circular dichroism spectrum of a 0.1 mg/g L-phenylalanine solution. (The maximum signal intensity was obtained at 216.5 nm). (**B**) The signal-to-noise ratio with different DIT times. (As the DIT time increased, the signal-noise ratio increased).

The bandwidth was set to 10 nm and the scan rate to 50 nm/min and then the detection wavelength and DIT were optimized for the maximum CD intensity and signal-to-noise ratio. Firstly, the detection wavelength was optimized using a 0.1 mg/g L-Phe solution. The solution was added into a 10 mm light path cuvette and it was scanned from 200 nm to 300 nm as shown in Fig. 2A. The maximum CD signal was obtained at a wavelength of 216.5 nm. Therefore, 216.5 nm was used as the optimal detection wavelength in the following experiment. Then the CD spectrum was scanned as the DIT was varied from 1 to 32 s in the Spectra software, and the signal-to-noise ratio of the L-Phe peak was calculated and compared as shown in Fig. 2B. As the DIT increased, though the intensity of the CD signal remained the same, the signal-to-noise ratio increased. To achieve maximum signal-to-noise, 32 s was chosen for the following experiment.

### Optical purity examination of the D-Phe

**Figure 3:**
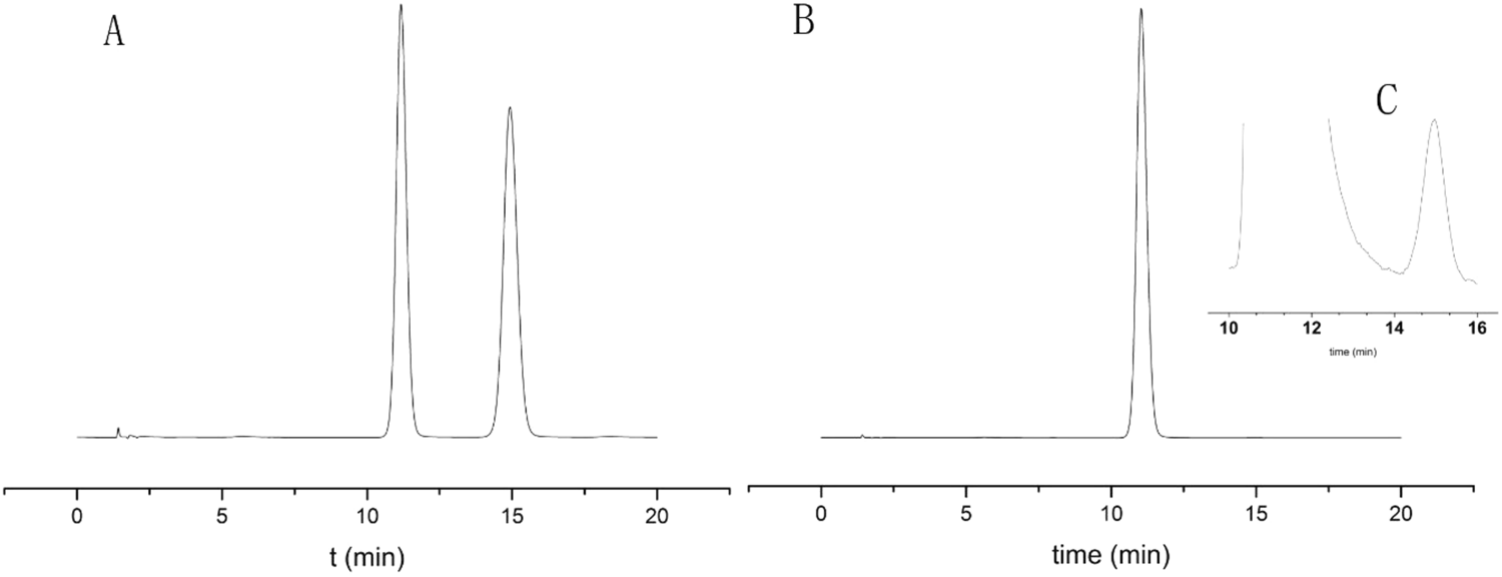
(**A**) Separation of equal amounts of D-phenylalanine and L-phenylalanine; (**B**) Purity assessment of D-phenylalanine. (**C**) Zoomed L-phenylalanine peak in D-phenylalanine.

The purity of D-Phe was firstly assessed by mass balance method, which was 99.25% (Supplementary Information). Then HPLC was used to examine the optical purity of D-Phe. The phenylalanine enantiomer mixture (0.05 mg/mL for each enantiomer) was separated by chiral column with a resolution of 7.54, confirming the successful separation (Fig. 3A). The chromatograph of pure D-Phe (0.1 mg/mL) is shown in Fig. 3B and indicates that only a trace amount of L-Phe was in the D-Phe. The D-Phe sample was analysed six times, and the peak areas of D- and L-Phe were used to calculate the L-Phe impurity in D-Phe (the L-Phe peak is shown in Fig. 3C), as shown in Table 1. The mass fraction of L-Phe in D-Phe was less than 0.1% with a RSD of 0.84% (Table 1). Therefore, the pure D-Phe sample was suitable to be used as the internal standard as it will not contribute a significant error to L-Phe quantitation.

**Table 1:** Mass fraction of L-phenylalanine in D-phenylalanine.

Sample	Peak area of D-Phe	Peak area of L-Phe	Content of L-Phe in D-Phe [%]
1	22466.9	15.7	0.070
2	22416.4	15.8	0.070
3	22421.9	16.0	0.071
4	22420.7	16.0	0.071
5	22475.8	15.8	0.070
6	22711.7	16.1	0.071
Average [%]	0.071		
RSD[%]	0.84		

### Evaluation of the method performance for pure L-phenylalanine quantitation

Firstly, the measurement principle was demonstrated with pure L-Phe solution as the samples and the method performance was evaluated. There are two approaches to quantify L-Phe in solution with D-Phe as the internal standard. In the first approach, the same amount of D-Phe was added into a series of L-Phe samples with different concentrations, therefore, a non-zero CD signal will be observed because of the different amounts of L-Phe and D-Phe in the solution. In the second approach, a different amount of the D-Phe was added into each individual L-Phe sample such that the amount of D-Phe was almost the same as that of L-Phe. Therefore, an almost zero CD signal will be observed. A series of simulated L-Phe samples were prepared from a L-Phe stock solution by gravimetric method covering the concentration range from 0.005 mg/g to 1 mg/g. Then the simulated samples were quantified by adding D-Phe as the internal standard using two approaches. The bracket calibration mode was used to quantify the L-Phe solution in each sample. For each concentration level, four replicates were analysed and the average was compared with that from the gravimetric value to calculate the bias and RSD.

As shown in Table 2, both approaches showed good accuracy and method variance in the concentration range from 0.04 mg/g to 0.8 mg/g. Both the bias and the RSD were less than 1% in this range, demonstrating good method accuracy and precision. The 3-fold and 10-fold of the signal from the pure solvent in the CD spectrum was used to calculate the limit of detection (LOD) and limit of quantitation (LOQ), which were1 μg/g and 4 μg/g, respectively.

**Table 2:** Method performance of L-phenylalanine quantitation by circular dichroism.

Fixed concentration of internal standard			Variable concentration of internal standard		
C_L-phe_ [mg/g]	Bias [%]	RSD [%]	C_L-phe_ [mg/g]	Bias [%]	RSD [%]
0.0049	−3.21	0.202	0.0049	2.85	2.203
0.0099	1.16	0.226	0.0099	2.24	0.596
0.0198	1.10	0.136	0.0198	0.82	0.264
0.0395	0.46	0.518	0.0396	0.73	0.266
0.0594	−0.59	0.386	0.0593	−0.25	0.182
0.0789	−0.62	0.157	0.0791	0.01	0.606
0.0990	−0.21	0.473	0.0990	−0.04	0.465
0.7940	−0.01	0.270	0.7919	0.27	0.481
0.9781	1.09	0.437	0.9899	2.15	5.468

### Optimization of HPLC separation condition

**Figure 4:**
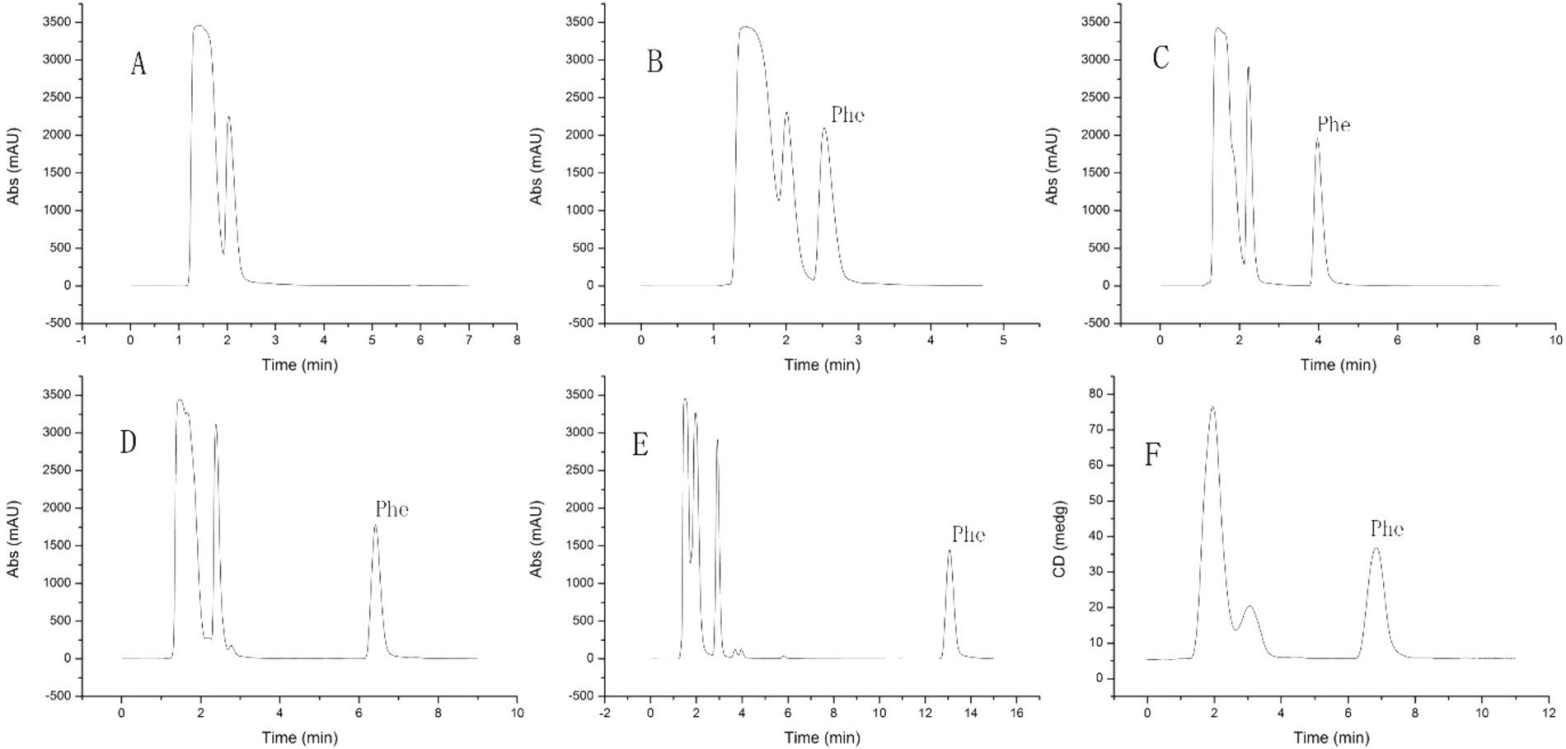
Separation of the L-phenylalanine from of the amino acid mixture using different mobile phases. (Water: Acetonitrile = (**A**) 80:20, (**B**) 90:10, (**C**) 95:5, (**D**) 98:2, (**E**) 100:0, DAD detector with a wavelength of 220 nm; (**F**) The CD spectrum of separation of amino acid mixture with CD detector using a mobile phase of A:B = 98:2; 0.5 mg/g of each amino acid).

After the demonstration of the measurement principle, CD detection combined with separation by HPLC should be realized. Therefore, the HPLC separation condition was firstly optimized to separate L-Phe from other amino acid mixture before the proposed method was used for real sample analysis. The amino acid mixture containing the common 15 amino acids was used to evaluate the separation efficiency of L-Phe. The separation of the amino acid mixture was carried out on the column (Waters UPLC HSS T3) with different mobile phase ratio of water:MeCN(A:B). Several different ratios of A:B (100:0, 98:2, 95:5, 90:10, 80:20) were compared for the separation. The DAD detector with a wavelength of 220 nm was used to monitor the separation of L-Phe. Finally, the ratio of A:B = 98:2 was applied in the separation of amino acid mixture in this study with a satisfactory separation within 10 min. The typical separation chromatogram is shown in Fig. 4, and the typical HPLC-CD spectrum of separation is shown in Fig. 4F.

### Evaluation of the method performance for quantification of L-phenylalanine in amino acid mixture

To evaluate the performance of quantification of L-phenylalanine in amino acid mixture, the amino acid mixture solution was analyzed by two approaches:fixed internal standard concentration and variable internal standard concentration, respectively. The bracket calibration mode was used for result calculation. For each concentration level, six replicates were analysed and the average was compared with its gravimetric value to calculate the bias and RSD, which are shown in Table 3. Both the bias and the RSD were less than 1% in the concentration range from 0.1 mg/g to 1.2 mg/g, demonstrating good method accuracy and precision. The 3-fold and 10-fold of the noise signal in the chromatogram was used to calculate the limit of detection (LOD) and limit of quantitation (LOQ), which were 4 μg/g and 30 μg/g, respectively.

**Table 3:** Method performance of L-phenylalanine quantitation by HPLC-CD.

Fixed concentration internal standard			Variable concentration internal standard		
C_L-phe_ [mg/g]	Bias [%]	RSD [%]	C_L-phe_ [mg/g]	Bias [%]	RSD [%]
0.0106	−0.699	3.753	0.0530	−0.711	2.815
0.0320	−0.870	1.761	0.0842	−1.038	1.898
0.0842	−0.783	1.147	0.1056	−0.398	1.750
0.1056	0.703	0.727	0.3129	0.393	1.820
0.3129	−0.257	0.628	0.5195	0.651	0.9374
0.5195	0.242	0.803	0.8292	−0.010	1.170
0.8292	0.330	0.697	1.0370	−0.264	1.392
1.0370	0.084	0.650			
1.0880	−0.856	0.844			
1.1874	0.811	0.773			
1.2799	0.792	1.327			
1.4823	−0.489	1.225			

### Evaluation of the method performance for quantification of the mass fraction of pINS in solid

**Figure 5:**
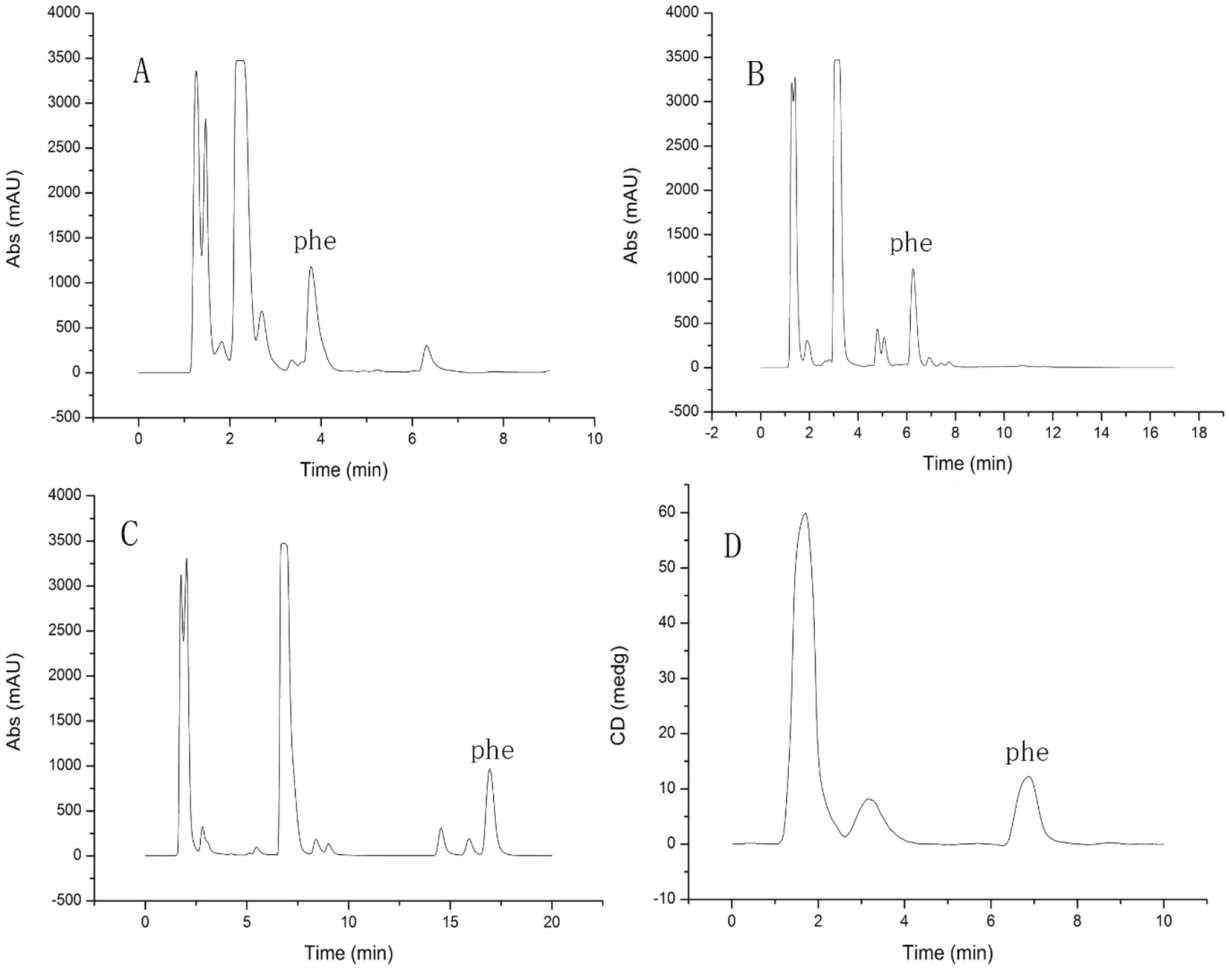
Separation of the L-phenylalanine from of the porcine insulin hydrolysate using different mobile phases. (Water: Acetonitrile = 95: 5 (**A**), 98:2 (**B**,**D**), 100:0 (**C**), DAD detector with a wavelength of 220 nm; (**D**): CD detector with a wavelength of 216.5 nm).

After validation of LC-CD method, it was used to determine the mass fraction of pINS in solid. Natural proteins consist of only L-amino acids, the mass fraction of pure protein could be determined by quantification of L-Phe after hydrolysis. The pINS CRM was chosen as the analyte. The solid pINS samples were hydrolysed to L-amino acid mixture, and analysed using the validated LC-CD method. Because some unstable amino acids were converted into other compounds during the hydrolysis, the pINS hydrolysate was not exactly the same of the amino acid mixture. Therefore, the HPLC separation condition was further optimized to separate L-Phe from others. The typical separation chromatogram was shown in Fig. 5.

The L-Phe in the hydrolysate was quantified using the fixed internal standard concentration approach and bracket calibration mode. The L-Phe concentration was firstly obtained by formula (13), then the mass fraction of pINS in the solid was calculated base on the amino acid composition of pINS according to formula (14).

14 $${c}_{INS}=\frac{{c}_{L}\times {M}_{INS}\times M\times m}{{m}_{sample}\times n\times {M}_{phe}\times {m}_{INS}}$$

where:

*c*_INS_ is the mass fraction of pINS, g/g.

*M*_phe_ and *M*_INS_ are the molecular weight of Phe and pINS, respectively.

*n* is the number of L-Phe residues in the pINS amino acid sequence.

*M* is the total mass of the hydrolysate (mixture of the sample solution, internal standard and hydrochloric acid in one ampoule), mg.

*m*_sample_ is the mass of the sample solution in 0.1 mol/L hydrochloric acid added in the ampoule, mg.

*m* is the total mass of solid pINS and 0.1 mol/L hydrochloric acid after dilution, mg.

*m*_INS_ is the mass of solid pINS, mg.

The same pINS sample was analyzed six times once resulting in an average mass fraction of 0.922 g/g, with a RSD of 0.89%, and showing good repeatability. Then the same pINS sample was analyzed three times a day over three consecutive days resulting in an average mass fraction of 0.923 g/g with a RSD of 1.9%, showing good reproducibility. The 3-fold and 10-fold of the noise signal in the chromatogram was used to calculate the limit of detection (LOD) and limit of quantitation (LOQ), which were 0.12 mg/g and 0.44 mg/g, respectively.

### Comparison with traditional IDMS method

The average mass fraction of pINS was 0.922 g/g, which was shown in Table 4. The uncertainty of pINS quantitation result was evaluated to be 0.035 g/g (*k* = 2, Supplementary Information). Therefore, the mass fraction of pINS determined by LC-CD was (0.922 ± 0.035) g/g. Compared with that obtained by IDMS of (0.892 ± 0.036) g/g^26^, the agreement was achieved by calculation of *E*_n_value.

15 $${E}_{n}=\frac{|{x}_{LC-CD}-{x}_{IDMS}|}{\sqrt{{U}_{LC-CD}^{2}+{U}_{IDMS}^{2}}}=\frac{|0{\rm{.922}}-{\rm{0}}\mathrm{.892}|}{\sqrt{{0.035}^{2}+0.0{{\rm{36}}}^{2}}}=0.80 < 1$$

**Table 4:** Quantification of the mass fraction of porcine insulin in solid by LC-CD [g/g].

Sample	1	2	3	4	5	6
Vial 1	0.9024	0.9226	0.9335	0.9051	0.9391	0.9029
Vial 2	0.9159	0.9208	0.9269	0.9281	0.9096	0.9312
Vial 3	0.9139	0.9397	0.9482	0.9032	0.9304	0.9267
Average	0.922					
RSD [%]	1.5					

Compared the proposed method with traditional IDMS method, it is found to have lots of similarities (Table 5), which makes the proposed method a potential primary method. The isotope labeled target is used as the internal standard for IDMS, and its physical and chemical properties are similar to that of the target. Therefore, the traditional IDMS method is a special kind of internal standard method. The related enantiomer of the target is used as the internal standard for the proposed method, and its physical and chemical properties are also similar to that of the target. Therefore, the proposed method is also a special kind of internal standard method. During the pretreatment process, the loss rate of target is similar to that of isotope labeled or enantiomer internal standard, because they have similar chemical and physical property. Therefore, the internal standards can correct the systematic and random error to the maximum extent. During the separation process, the isotope labeled internal standard has the similar retention time as the target, therefore the same random interference will be applied in both the internal standard and the target. The same will occur when an enantiomer is used as the internal standard in a non-chiral separation process. For IDMS method, ionization is required and both target and internal standard have a similar ionization rate so as to reduce the systematic and random error. No ionization is required for the proposed method and potentially leads a more accurate result. The IDMS method is applied with mass spectrometry in a vacuum condition and regulated by ion optical system. However, the proposed method is applied with CD spectrometry in a normal condition and regulated by optical system, which tends to be more stable and leads to a more accurate and precise result. For both methods, the single point, bracket and standard curve calibration mode could be applied with flexibility. The similar behavior of the proposed method makes it a potential primary method compared with traditional IDMS method.

**Table 5:** Comparison of the proposed method with traditional IDMS method.

Method	Principle	Internal standard	Pretreatment	Separation	Ionization	Regulation	Detection	Calibration
HPLC-IDMS	Internal standard with isotope labeled target	Similar physical and chemical property as the target	Similar loss rate as the target	Similar retention time	Smilar ionization rate	Ion optical system, vacuum needed	Mass spectrom-etry	Single point, bracket, or standard curve
HPLC-CD	Internal standard with related enantiomer	Similar physical and chemical property as the target	Similar loss rate as the target	Similar retention time	No ionizati-on required	Optical system, no vacuum required	CD spectrom-etry	Single point, bracket, or standard curve

### Scope and merit of the proposed method

The proposed method can be applied in the quantification of L-amino acids with a high concentration directly, therefore, it can be used for amino acid mixture analysis. It can be also used for peptide or protein purity analysis because a higher concentration can be achieved for the purity analysis. And it is expected to be potentially used for chiral compound quantitation in high concentration in matrix, for example, for bio-active compound quantitation of traditional Chinese medicine. The proposed method has several advantages and merits as following: firstly, the proposed method is based on optical spectrometry and no ionization or ion control is needed. Compared with mass spectrometry technology, optical signal is more stable. Therefore, the proposed method is expected to have better repeatability, which will result in a lower uncertainty. Secondly, some amino acids have low UV absorbance and these cannot be quantified by UV-Vis detector directly without any derivation. However, these amino acids show better CD signal and they can be quantified directly without derivation with this method. Thirdly, the measurement principles can be totally described by equations and understood, which makes it to be a potential primary method. Though it can be only applied in a few occasion, it is still a useful complementary method for traditional IDMS method. Finally, the samples can be re-used after the analysis by fraction collection, and the cost is less compared with IDMS because no isotopic compound is used.

## Conclusion

A novel potential primary method for enantiomer quantitation based on high-performance liquid chromatography-circular dichroism is described using L-phenylalanine as an example in this study. The measurement principles are illustrated in detail and the method performance were demonstrated to have good accuracy and repeatability. The proposed method can be entirely described and understood by equations and a complete uncertainty statement can be defined in SI units. Therefore, it may be a potential primary method useful for the quantification of chiral compounds and proteins with a high concentration, and a supplementary method to the traditional isotope dilution mass spectrometry approach. Please be aware that the proposed method cannot solve the hydrolysis problem for peptide or protein purity assessment. When the proposed method is used, the hydrolysis should be investigated carefully in order to achieve an accurate result.

## Electronic supplementary material

Supplementary material
