# Supplementary material for: A novel potential primary method for quantification of enantiomers by high performance liquid chromatography-circular dichroism
Source: Sci Rep. 2018 May 9;8:7390. doi: 10.1038/s41598-018-25682-4 (PMC5943587; doi:10.1038/s41598-018-25682-4)
Supplement: Supplementary file 1 — Supplementary material [file 41598_2018_25682_MOESM1_ESM.docx]

**A novel potentialprimary method for quantification of enantiomers by high performance liquid chromatography-circular dichroism**

**Yi LUO^1^, Liqing WU^1*^, Bin YANG^1^, Youxun JIN^1^, Kangle ZHENG^2^, Zhangjing HE^2^**

^1^National Institute of Metrology, P.R. China, Beijing, China

^2^Beijing University of Chemical Technology, Beijing, China

^*^Authors for correspondence

E-mail: wulq@nim.ac.cn

National Institute of Metrology, P.R. China, No. 18Beisanhuan East Road, Chaoyang District, Beijing, China

**Supplementary Information**

**1.Purity assessment of the D-Phenylalanine**

**1.1 Instruments and reagents**

The electronic balances, Mettler Toledo XP56 (0.001 mg) was purchased from Mettler Toledo (Zurich, Switzerland). The high-performance liquid chromatography system (HPLC, Aglient 1260 Infinity), equipped with an autosampler, quaternary pump, column compartment, and PDA detector (Agilent 1290 Infinity), were purchased from Agilent Technologies (Santa Clara, CA, USA). The Daicel CROWNPAK CR + column (4.0 mm ×150 mm, 5 μm) was purchased from Daicel Corporation (Tokyo, Japan). The gas chromatography mass spectrometer (GC/MS), equipped with an autosampler (TriPlus RSH), gas chromatography (Trace GC Ultra) and mass spectrometer (ISQ MS), were purchased from Thermo Fisher Scientific (Waltham, MA, USA). The analytical column was J&W VF-624ms (0.25 mm × 30 m, 1.40 μm) purchased from Aglient Technologies (Santa Clara, CA, USA). The muffle furnace (KDF-S80) was purchased from DENKEN Corporation (Kyoto, Japan).

D-phenylalanine (D-Phe) was purchased from Sinopharm Chemical Reagent Co., Ltd (Shanghai, China). The perchloric acid (A.R. grade) was purchased from Beijing Chemworks (Beijing, China).

**1.2 Method**

The mass balance method was used for purity assessment of the D-phenylalanine. The moisture, organic impurities, volatile organic compounds (VOCs), inorganic compounds were measured respectively. Then the purity of D-phenylalanine was estimated by formula 1.

$\omega=P\times(1-A-B-C)$ 1

where,

$\omega$is the purity of the D-phenylalanine, %;

*P* is the HPLC purity of the D-phenylalanine, %;

*A* is the mass fraction of the moisture, %;

*B* is the mass fraction of the ignition residues , %;

*C* is the mass fraction of the VOCs, %.

The HPLC purity analysis was performed using a chiral column (CROWNPAK CR+, 4.0 mm×150 mm). A perchloric acid aqueous solution at a pH of 1.55 was used as the mobile phase with a flow rate of 0.8 mL/min. The injection volume was 10 μL and the detection wavelength was 200 nm. The HPLC

purity was determined by the area ratio of the main peak to all peaks.

The amount of the moisture was estimated by the loss of weight at 105 ºC. The weighing bottle was firstly put into the oven at 105 ºC. The weight was measured every 1 hr until a constant weight was obtained. Then fifty milligram of D-phenylalanine was put into the weighing bottle and dried in the oven at 105ºC. The weighing bottle containing the sample was weighed every 1 hr until a constant weight was obtained. Then the loss of the weight was used to calculate the moisture mass fraction.

The amount of inorganic compounds was estimated by the ignition residue at 800 ºC in the muffle furnace. The crucible was cleaned and put into the muffle furnace at 800ºC for 5 hr. The weight of the crucible was measured and the ignition process was repeated until a constant was obtained. Then 150 mg D-phenylalanine was weighed into the crucible and the ignition process was repeated again until a constant was obtained finally. 150 mg into 3 empty clean burned and weighted by balance before. The samples were heating at 800ºC The residue weight was used calculated the mass fraction of the ignition residue.

The mass fraction of VOCs was determined by GC-MS. One hundred milligram of D-phenylalanine was put into the headspace vials and equilibrated overnight. Then the GC-MS analysis was performed by headspace injection. During the injection process, the headspace vials was firstly put into an incubator at 80ºC for 30 min. The inject temperature was 90ºC. The SSL inlet was operated at 200ºC in split mode with the split ratio of 10:1. The GC carrier gas was helium with a flow rate of 1.0 mL/min. The initial oven temperature was started at 32ºC and hold for 2.5 min, then to 45ºC at a ramping rate of 2ºC/min, next to 180ºC with a ramping rate of 30ºC/min and hold for 0.5 min. EI was used for ionization and the MS transfer line temperature was set to 280ºC. The ion source temperature was set to 230ºC. The detector was set with the scan range from 20 to 150 amu.

**1.3 Results**

The D-phenylalanine sample was analyzed for six times and the HPLC purity results were 99.56%, 99.63%, 99.68%, 99.66%, 99.68%, 99.63%, respectively. An average of 99.64% was used for calculation. Three individual D-phenylalanine samples were taken for moisture analysis and the results were 0.3%，0.21% and 0.16%, respectively. An average of 0.22% was used for calculation. Also, three individual D-phenylalanine samples were taken for ignition residue analysis and the results were 0.27%，0.13%，0.11%, respectively. An average of 0.17% was used for calculation. Two individual D-phenylalanine samples were taken for VOCs analysis and each sample was analyzed for three time. No obvious peak was detected by GC-MS. Therefore, the VOCs was estimated as zero. The typical ion chromatogram of GC-MS was shown in Figure S1. The final purity of D-phenylalanine was calculated by formula 1, which was 99.25%.


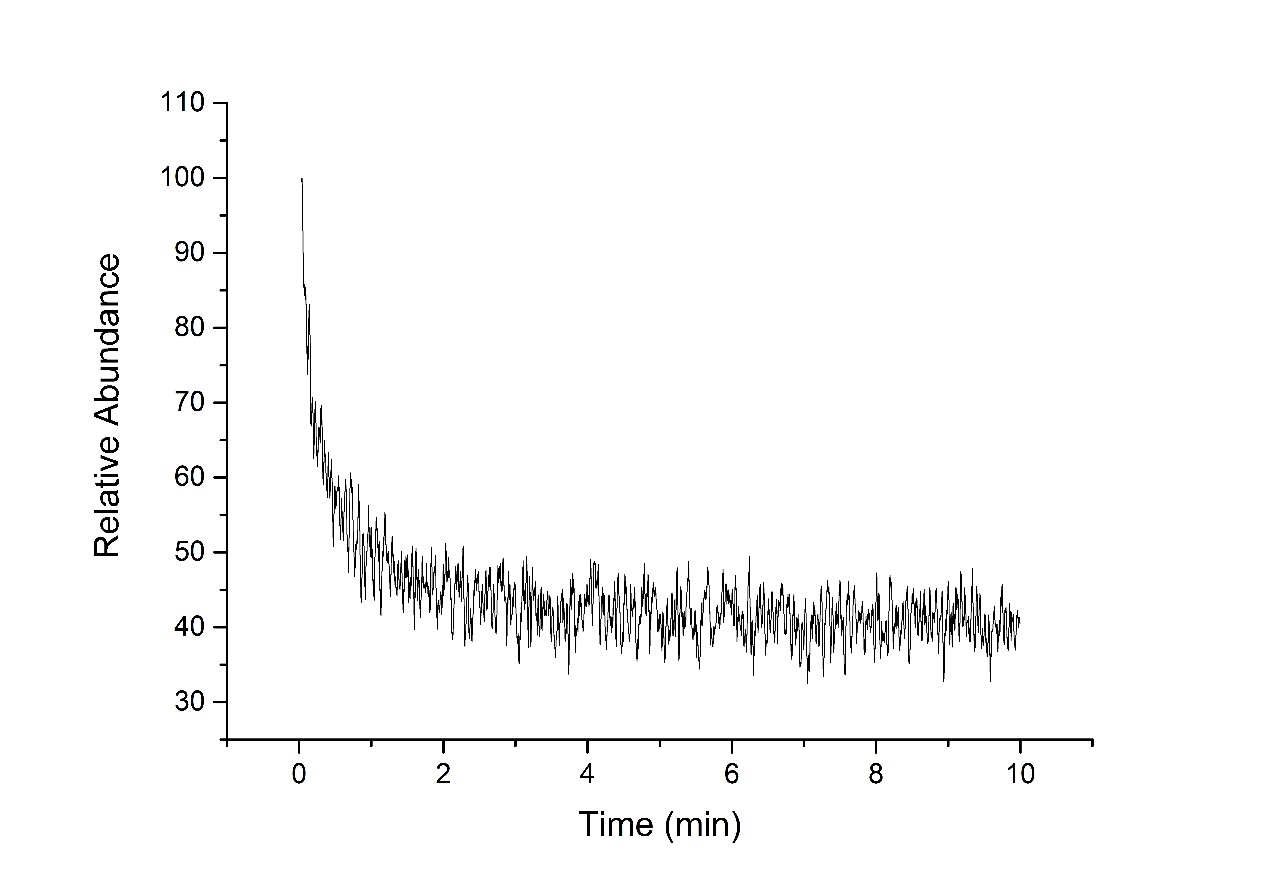


Figure S1 Typical ion chromatogram of GC-MS by headspace injection

**2. Uncertainty evaluation of pINS quantitation result**

Three pINS samples were analysed six times each. The average was 0.922 g/g with a RSD of 1.5% as shown in Table 4. Then the associated uncertainty of the average was evaluated. According to the measurement steps, the uncertainty mainly came from the weighing process, hydrolysis efficiency, and repeatability of the method and the uncertainty of the amino acid standard.

The balance imprecision with a rectangle distribution was used to evaluate the uncertainty for each weighing process. The balance imprecision used for phenylalanine and water weighing were 0.001 mg and 0.00001 g, respectively. Therefore, the uncertainty caused by weighing L-Phe and water were showed in Supplementary Discussion. The illustration of the symbols used for the uncertainty evaluation was listed in Supplementary TableS1.

$$\mu_{w,Phe}=\frac{0.001}{\sqrt{3}}=0.000577\text{mg}$$

$$\mu_{w,Water-Phe}=\frac{0.00001}{\sqrt{3}}=5.77\times10^{-6}g$$

According to certificate of L-Phe CRM, the purity was (99.4±1.5)%, *k*=2, indicating the standard uncertainty was 0.75%.

$$\mu_{P,L-phe}=\frac{U_{Phe}}{k}=\frac{1.5\%}{2}=0.75\%$$

The uncertainty from the purity of D-Phe was calculated by the standard deviation of 6 times purity analyses.

$$\mu_{P,D-Phe}=s_{D-Phe}=0.84\%$$

The stock solution was prepared by solid L-Phe gravimetrically, its concentration could be calculated by formula3. Therefore, the uncertainty of the L-Phe working solution was as following:

$m_{s,L-Phe}=m_{m,L-Phe}\text{+}m_{water,L-Phe}$ 2

$$\mu_{s,L-Phe}=\sqrt{\mu_{m,L-Phe}^{2}+\mu_{water,L-Phe}^{2}}=\sqrt{(5.77\times10^{-7})^{2}+(5.77\times10^{-6})^{2}}=5.8\times10^{-6}\text{g}$$

$c_{L-Phe}=\frac{m_{m,L-Phe}\cdot P_{L-Phe}}{m_{s,L-Phe}}$ 3

$$\begin{matrix} \mu_{c,L-Phe}=c_{L-Phe}\cdot\sqrt{\left(\frac{\mu_{m,L-Phe}}{m_{m,L-Phe}}\right)^{2}+\left(\frac{\mu_{s,L-Phe}}{m_{s,L-Phe}}\right)^{2}+\left(\frac{\mu_{P,L-Phe}}{P_{L-Phe}}\right)^{2}} \\ =0.506962\times\sqrt{\left(\frac{0.000577}{6.408}\right)^{2}+\left(\frac{5.80\times10^{-6}}{12.564168}\right)^{2}+\left(\frac{0.75\%}{99.4\%}\right)^{2}}=0.003825\text{mg/g} \end{matrix}$$

The uncertainty of the concentration of D-Phe working solution was calculated as following:

$c_{D-Phe,WS}=\frac{c_{D-Phe,stock}\cdot m_{D-Phe,stock}}{m_{s,D-Phe,WS}}$ 4

$$\begin{matrix} \mu_{c,D-Phe,WS}=c_{D-Phe,WS}\cdot\sqrt{\left(\frac{\mu_{c,D-Phe,stock}}{c_{c,D-Phe,stock}}\right)^{2}+\left(\frac{\mu_{m,D-Phe,stock}}{m_{D-Phe,stock}}\right)^{2}+\left(\frac{\mu_{s,D-Phe,WS}}{m_{s,D-Phe,WS}}\right)^{2}} \\ =0.049156\times\sqrt{\left(\frac{0.004196}{0.496974}\right)^{2}+\left(\frac{5.77\times10^{-6}}{0.98996}\right)^{2}+\left(\frac{5.77\times10^{-6}}{10.00863}\right)^{2}}=0.000415\text{mg/g} \end{matrix}$$

The lower and higher standard solution were both mixed by L-Phe and D-Phe working solution. The uncertainty of the concentration of L-Phe in the lower standard was calculated as following:

$m_{1}=m_{L-Phe,1}+m_{D-Phe,1}$ 5

$$\mu_{m,1}=\sqrt{\mu_{m,L-Phe,1}^{2}+\mu_{m,D-Phe,1}^{2}}=\sqrt{0.000577^{2}+0.000577^{2}}=0.000816\text{mg}$$

$c_{L1}=\frac{m_{L-Phe,1}\cdot c_{L-Phe}}{m_{1}}$ 6

$$\begin{matrix} \mu_{cL1}=c_{L1}\cdot\sqrt{\left(\frac{\mu_{m,L-Phe,1}}{m_{L-Phe,1}}\right)^{2}+\left(\frac{\mu_{m,1}}{m_{1}}\right)^{2}+\left(\frac{\mu_{c,L-Phe}}{c_{L-Phe}}\right)^{2}} \\ =0.168965\times\sqrt{\left(\frac{0.000577}{200.297}\right)^{2}+\left(\frac{0.000816}{600.969}\right)^{2}+\left(\frac{0.003825}{0.506962}\right)^{2}}=0.001275\text{mg/g} \end{matrix}$$

Similarly, the uncertainty of the concentration of L-Phe in higher standard, D-Phe in lower and higher standards were 0.001913 mg/g, 0.000207 mg/g and 0.000207 mg/g, respectively. The uncertainty of theconcentration of D-Phe in hydrolysate was as calculated as following:

$M=m_{sample}+m_{D-Phe,IWS}+m_{HCl}$ 7

$$\mu_{M}=\sqrt{\mu_{m,sample}^{2}+\mu_{m,D-Phe,IWS}^{2}+\mu_{m,HCl}^{2}}=\sqrt{0.000577^{2}\times3}=0.001\text{mg}$$

$c_{D}=\frac{c_{D-Phe,IWS}\cdot m_{D-Phe,IWS}}{M}$ 8

$$\begin{matrix} \mu_{cD}=c_{D}\cdot\sqrt{\left(\frac{\mu_{c,D-Phe,IWS}}{c_{D-Phe,IWS}}\right)^{2}+\left(\frac{\mu_{M}}{M}\right)^{2}+\left(\frac{\mu_{m,D-Phe,IWS}}{m_{D-Phe,IWS}}\right)^{2}} \\ =0.009094\times\sqrt{\left(\frac{0.001677}{0.198648}\right)^{2}+\left(\frac{0.001}{1077.498}\right)^{2}+\left(\frac{0.000577}{49.328}\right)^{2}}=7.68\times10^{-5}\text{mg/g} \end{matrix}$$

According to formula 13 described in the article:

$c_{L}=\frac{(I_{2}-I)(c_{L1}-c_{D1})+(I-I_{1})(c_{L2}-c_{D2})}{(I_{2}-I_{1})}+c_{D}$ 9

The uncertainty of the concentration of L-Phe in hydrolysis solution was following:

$$\begin{matrix} \mu_{cL}=\sqrt{\left(\frac{(I-I_{1})\cdot\mu_{c2}}{I_{2}-I_{1}}\right)^{2}+\left(\frac{(I_{2}-I)\cdot\mu_{c1}}{I_{2}-I_{1}}\right)^{2}+\mu_{cD}^{2}} \\ =\sqrt{\left(\frac{(6.8609-5.11443)\times0.001924}{8.6347-5.11443}\right)^{2}+\left(\frac{(8.6347-6.8609)\times0.001305}{8.6347-5.11443}\right)^{2}+\left(7.68\times10^{-5}\right)^{2}}=0.001162\text{mg/g} \end{matrix}$$

Therefore, the type B uncertainties were combined together finally.

$K=\frac{M_{INS}}{M_{phe}\cdot n}=\frac{5777.6}{165.19\times3}=11.6585$ $\mu_{sample}=\sqrt{\left(\frac{K\cdot c_{L}\cdot m}{m_{\text{sample}}\cdot m_{INS}}\mu_{M}\right)^{2}+\left(\frac{K\cdot c_{L}\cdot M}{m_{sample}\cdot m_{INS}}\mu_{m}\right)^{2}+\left(\frac{K\cdot m\cdot M}{m_{sample}\cdot m_{INS}}\mu_{cL}\right)^{2}+\left(\frac{K\cdot c_{L}\cdot m\cdot M}{m_{sample}^{2}\cdot m_{INS}}\mu_{m,sample}\right)^{2}+\left(\frac{K\cdot c_{L}\cdot m\cdot M}{m_{sample}\cdot m_{INS}^{2}}\mu_{mINS}\right)^{2}}=0.00563\text{g/g}$

The uncertainty of the method reproducibility was calculated by the relative standard deviation of 18-repeatanalyses:

$$\mu_{method}=s\cdot c_{INS}=1.5127\%\times0.9222=0.01395\text{g/g}$$

The uncertainty of the hydrolysis efficiency was estimated as 1% of the average. Therefore, the standard uncertainty was obtained by combining all the uncertainty components:

$$\mu_{c}=\sqrt{\mu_{sample}^{2}+\mu_{hydrolysis}^{2}+\mu_{method}^{2}}=\sqrt{0.00563^{2}+0.00922^{2}+0.01395^{2}}=0.01764\text{g/g}$$

The expanded uncertainty was calculated as following with a coverage factor of 2:

$$U=2\mu_{c}=2\times0.01764=0.035\text{g/g}$$

Table S1 Illustration of the symbol used in uncertainty evaluation

| μ_w,Phe_ | 0.000577 mg | The uncertainty caused by weighing L-Phe. |
| --- | --- | --- |
| μ_w,water-Phe_ | 5.77×10^-6^ g | The uncertainty caused by weighing water. |
| μ_P,L-Phe_ | 0.75% | The uncertainty of the purity of L-Phe CRM. |
| U_Phe_ | 1.5% | Expanded uncertainty of L-Phe CRM. |
| μ_P,D-Phe_ | 0.84% | The uncertainty caused by weighing D-Phe. |
| s_D-Phe_ | 0.84% | Standard deviation of D-Phe purity analysis. |
| m_s,L-Phe_ | 12.564168 g | Total weight of L-Phe solution. |
| m_m,L-Phe_ | 6.408 mg | Weight of solid L-Phe CRM in preparation of L-Phe solution. |
| m_water,L-Phe_ | 12.55776 g | Weight of water in preparation of L-Phe solution. |
| μ_s,L-Phe_ | 5.80×10^-6^ g | The uncertainty of the total weight of L-Phe solution. |
| μ_m,L-Phe_ | 0.000577 mg | The uncertainty caused by weighing solid L-Phe CRM in L-Phe solution. |
| μ_water,L-Phe_ | 5.77×10^-6^ g | The uncertainty caused by weighing water in L-Phe solution. |
| c_L-Phe_ | 0.506962 mg/g | The concentration of L-Phe solution. |
| P_L-Phe_ | 99.4% | The purity of L-Phe CRM. |
| μ_c,L-Phe_ | 0.003825 mg/g | The uncertainty of the concentration of L-Phe solution. |
| c_D-Phe,WS_ | 0.049156 mg/g | The concentration of D-Phe working solution. |
| c_D-Phe,stock_ | 0.496974 mg/g | The concentration of D-Phe stock solution. |
| m_D-Phe,stock_ | 0.98996 g | Weight of D-Phe stock solution in preparation of D-Phe working solution. |
| m_s,D-Phe,WS_ | 10.00863 g | Total weight of D-Phe working solution. |
| μ_c,D-Phe,WS_ | 0.000415 mg/g | The uncertainty of the concentration of D-Phe working solution. |
| μ_c,D-Phe,stock_ | 0.004196 mg/g | The uncertainty of the concentration of D-Phe stock solution. |
| μ_m,D-Phe,stock_ | 5.77×10^-6^ g | The uncertainty caused by weighing D-Phe stock solution in preparation of D-Phe working solution. |
| μ_s,D-Phe,WS_ | 5.77×10^-6^ g | The uncertainty of the total weight of D-Phe working solution. |
| m_1_ | 600.969 mg | Total weight of Lower standard solution. |
| m_L-Phe,1_ | 200.297 mg | Weight of L-Phe solution in preparation of lower standard solution. |
| m_D-Phe,1_ | 400.672 mg | Weight of D-Phe working solution in preparation of lower standard solution. |
| μ_m,1_ | 0.000816 mg | The uncertainty of the total weight of lower standard solution. |
| μ_m,L-Phe,1_ | 0.000577 mg | The uncertainty caused by weighing L-Phe solution in preparation of lower standard solution. |
| μ_m,D-Phe,1_ | 0.000577 mg | The uncertainty caused by weighing D-Phe working solution in preparation of lower standard solution. |
| c_L1_ | 0.168965 mg/g | The concentration of L-Phe in lower standard solution. |
| μ_cL1_ | 0.001275 mg/g | The uncertainty of the concentration of L-Phe in lower standard solution. |
| c_1_ | 0.136193 mg/g | The difference between the concentration of L- and D-Phe in lower standard solution. |
| c_D1_ | 0.032773 mg/g | The concentration of D-Phe in lower standard solution. |
| μ_c1_ | 0.001305 mg/g | The uncertainty of the difference between the concentration of L- and D-Phe in lower standard solution. |
| μ_cD1_ | 0.000277 mg/g | The uncertainty of the concentration of D-Phe in lower standard solution. |
| M | 1077.498 mg | Total weight of hydrolysis solution. |
| m_sample_ | 197.657 mg | Weight of sample solution in preparation of hydrolysis solution. |
| m_D-Phe,IWS_ | 49.328 mg | Weight of D-Phe internal working solution in preparation of hydrolysis solution. |
| m_HCl_ | 830.513 mg | Weight of 8 mol/L HCl in preparation of hydrolysis solution. |
| μ_M_ | 0.001 mg | The uncertainty of total weight of hydrolysis solution. |
| μ_m,sample_ | 0.000577 mg | The uncertainty caused by weighing sample solution in preparation of hydrolysis solution. |
| μ_m,D-Phe,IWS_ | 0.000577 mg | The uncertainty caused by weighing D-Phe internal working solution in preparation of hydrolysis solution. |
| μ_m,HCl_ | 0.000577 mg | The uncertainty caused by weighing 8N HCl in preparation of hydrolysis solution. |
| c_D_ | 0.009094 mg/g | The concentration of D-Phe in the hydrolysis solution. |
| c_D-Phe,IWS_ | 0.198648 mg/g | The concentration of D-Phe internal working solution. |
| μ_cD_ | 7.68×10^-5^ mg/g | The uncertainty of the concentration of D-Phe in hydrolysis solution. |
| μ_c,D-Phe,IWS_ | 0.001677 mg/g | The uncertainty of the concentration of D-Phe internal working solution. |
| μ_cL_ | 0.001162 mg/g | The uncertainty of the concentration of L-Phe in hydrolysis solution. |
| I | 6.8609 medg·min | Peak area of hydrolysis solution. |
| I_1_ | 5.11443 medg·min | Peak area of lower standard solution. |
| I_2_ | 8.6347 medg·min | Peak area of higher standard solution. |
| μ_c2_ | 0.001924 mg/g | The uncertainty of the difference between the concentration of L- and D-Phe in higher standard solution. |
| m | 1.011007 g | Total weight of the sample solution. |
| m_INS_ | 13.263 mg | Weight of solid pINS in the sample solution. |
| m_HCl,INS_ | 0.997744 g | Weight of 0.1N HCl solution in the sample solution. |
| μ_m_ | 5.80×10^-6^ g | The uncertainty of total weight of the sample solution. |
| μ_m,INS_ | 5.77×10^-7^ g | The uncertainty caused by weighing solid pINS in sample solution. |
| μ_m,HCl,INS_ | 5.77×10^-6^ g | The uncertainty caused by weighing 0.1N HCl in preparation of sample solution. |
| K | 11.6585 | A constant. |
| M_INS_ | 5777.6 | The molecular weight of pINS. |
| M_phe_ | 165.19 | The molecular weight of phenylalanine. |
| n | 3 | The number of L-Phe residue in one pINS molecule. |
| μ_sample_ | 0.00563 g/g | The combined uncertainty of the type-B uncertainties. |
| c_L_ | 0.191292 mg/g | The concentration of L-Phe in hydrolysis solution. |
| μ_method_ | 0.01395 g/g | The uncertainty of method reproducibility. |
| s | 1.5127% | The relative standard deviation of 18-repeat analyses. |
| c_INS_ | 0.9222 g/g | The mass fraction of pINS in solid. |
| μ_c_ | 0.01764 g/g | The combined standard uncertainty. |
| μ_hysrolysis_ | 0.00922 g/g | The uncertainty of hydrolysis efficiency. |
| U | 0.03528 g/g | The expanded uncertainty. |
